# Welfare Assessment of Dairy Cows in Small Farms in Bangladesh

**DOI:** 10.3390/ani10030394

**Published:** 2020-02-28

**Authors:** M. Ariful Islam, Arvind Sharma, S. Ahsan, S. Mazumdar, K.C Rudra, Clive J.C. Phillips

**Affiliations:** 1 Centre for Animal Welfare and Ethics, School of Veterinary Science, The University of Queensland, Gatton 4343, Australia; maislam77@bau.edu.bd (M.A.I.);; 2 Department of Medicine, Faculty of Veterinary Science, Bangladesh Agricultural University, Mymensingh 2202, Bangladesh; shamirahsan2@gmail.com (S.A.); shimuldvm@gmail.com (S.M.);

**Keywords:** body condition score, milk yield, welfare assessment, dairy cow, Bangladesh

## Abstract

**Simple Summary:**

Welfare assessment is common in large, intensive dairy farms in the developed world, but has been little explored in small dairy farms in developing countries. We utilized and modified parts of an assessment protocol used for large farms to survey hunger, injuries, and disease as important components of the welfare of dairy cows in 70 small farms in Bangladesh. The incidence of body lesions was relatively high, but few cows were lame, compared with most surveys of intensive dairy farms in developed countries. Cows with common dairy diseases were thinner and produced less milk. Few farmers understood the concept of animal welfare and most did not vaccinate their cows against common diseases. We conclude that there were some similar problems in the Bangladeshi farms to those observed in large, intensive dairy farms, but also some differences. We suggest that it would be beneficial to improve the floors, bedding, and health status of cows, which would both increase their productivity and welfare.

**Abstract:**

Protocols for assessing the welfare of dairy cows in large scale intensive dairy systems in the developed world have been used extensively. Little attention has been paid to the use of similar welfare assessment protocols for small dairy enterprises in developing countries. We modified part of the standard assessment protocol and used it to assess aspects of the welfare of dairy cows in a field survey of 70 small farms in the Sirajgonj district of Bangladesh. Welfare indicators selected were mainly those of health and economic importance, such as lameness, lesions on the body and limbs, cleanliness levels, milk yield, and body condition. The study included physical examination of 700 cows and use of a structured questionnaire to collect data on health and management practices and farmers’ perspectives about animal welfare. Mean milk yield, averaged across farms, was 10.3 L/d (range 6.3–14.2) and body condition assessment indicated that cows were, on average, thin. Hygiene management was often poor, with soiling of body parts with faeces. The prevalence of lameness, at 4.3%, was less than has commonly been observed in larger, more intensive dairy farms, but body injuries were commonly detected at the carpal and hock joints (56 and 51% of cows, respectively). This suggests that floors and/or bedding to lie on were inadequate. Many farmers did not follow routine vaccination and deworming schedules (63% and 31%, respectively) and farmers were not generally aware of the concept of animal welfare. The study demonstrates some similar welfare issues to those that have been commonly identified in large, intensive units, but also some differences, in particular a failure to provide good floors, bedding, and basic health care.

## 1. Introduction

The welfare of dairy cows is a major concern of the public in developed countries [1], where consumers are increasingly oriented toward buying products from animals whose welfare is not compromised, and where it is guaranteed that products are in line with the standards of good agricultural practice [2]. Even though farmers are concerned about the condition of their animals [3], to meet growing public demand for high welfare, they must adapt their management practices in order to improve and optimize the welfare of their herd. Previously, animal welfare was understood to relate just to major concerns, such as serious hunger, thirst, injuries, or illness. For many years, welfare considerations have included discomfort, distress, fear, pain, and absence of normal behaviour [4]. It is now expanded to a multidimensional concept that includes physical and mental health, the absence of hunger, and provision for a manifestation of the typical behaviour for that species [5]. 

Assessment of dairy cow welfare is possible by a variety of methods. Four fundamental principles on which to base an integrated welfare assessment are good feeding, housing, health status and behaviour [6]. Inadequate housing and feeding expose cattle to numerous stressors and unpleasant emotions, which correspondingly affect their immunity, disease status, and behavioural disorders. Many researchers have developed methods for estimation of the welfare of cattle on farms [7,8,9], most of which include animal-related parameters, such as behaviour, body condition score (BCS), body cleanliness, lameness, skin lesions, injuries, and swellings. On-farm assessment of animal welfare can also be based on an evaluation of the provision of resources and management, direct observation of the animals and examination of farm records [10]. Associations have been reported between BCS, body weight change and fertility [11,12], health [13,14], and milk production [15,16,17], but these are not well understood for the zero-grazing systems predominating in developing countries. 

In relation to health, common welfare indicators are mortality, injury, productivity, and physiological and behavioural disturbances [18,19]. Risk factors for the main health disorders (mastitis, lameness, and metabolic disorders) afflicting dairy cows have been evaluated. Cows are commonly exposed to mud and faeces, but they avoid contaminated areas if they can [20]. There is an association between dirtiness of cows and their susceptibility to gastrointestinal problems [21], mastitis [22], and digital dermatitis [23]. The dirtiness of cows, especially of the hind limbs and udders, is also affected by stall design and length of chains, barn design, bedding, scraping frequency, stocking density, and lying times [24,25]. Coat contamination with faeces and urine can lead to irritation of the skin and discomfort to the animal [26]. Acute welfare change can be indicated by a decrease in productivity, in particular milk yield, but may also be evidenced by development of illness or injury, and changes in behaviour, for animals that are lethargic, unwilling to move, or unusually aggressive [27]. 

On-farm assessment of animal welfare is based on the evaluation of the provision of resources and management, direct observation of the animals, and the examination of farm records [10]. Whereas inspection of the environment may indicate the potential for certain welfare conditions, the assessment of the animals’ health, body condition, and behavior can be used to directly infer the effects of housing and management on the welfare of these animals. 

Animal welfare can, therefore, be inferred by several methods: behavioural, physiological, psychopathological assessments, longevity, and productive performances. All the welfare indicators have, to a greater or lesser extent, constraints and are unreliable when used as sole assessment techniques. It is widely acknowledged that better results can be obtained by measuring animal welfare using a range of indicators instead of individual parameters, e.g., Winckler et al., [28]. 

The increasing attention towards animal welfare has resulted in the formulation of many different welfare assessment protocols. The Welfare Quality protocol [6], developed in the European Union, is one of the best-known protocols for welfare assessment. It aims to evaluate the feeding, health, housing and behaviour of the cattle, recognizing that the most important parameters include hunger, thirst, resting, thermal comfort, ease of movement, absence of pain, injury and disease, ability to express social and other behaviours, a good human–animal relationship, and a positive emotional state. It bases the assessment of dairy cow welfare on comfort, disease, production, and cleanliness parameters. Methods of assessment in any region need to be tailored to the systems utilized for dairy farming. Cleanliness, integument alterations, lameness, and milk yield recur as important factors contributing to the welfare of the cattle or indicating their general condition across systems [6,29]. 

Small-holder dairy farms are the backbone of the rural economy in many developing countries, supporting the livelihoods of many farmers of low socioeconomic status. They play a significant role in poverty alleviation and reduction of malnutrition. The dairy cattle in these farms are a regular source of income, provide employment and cater to the protein needs of the rural population [30]. Small holder dairy farming suffers from constraints—such as diseases, deficient management, and inadequate feeding practices—that compromise the welfare of cattle reared in such systems [31]. Acceleration in the intensification of small holder systems with the aim of increasing the income of this disadvantaged sector of the population is likely to affect the welfare of the dairy cows in such systems. The developing western concept of intensive dairying has some specific welfare disadvantages, in particular lack of outdoor access. Pasture access generally increases cow health and comfort and reduces aggression between cows, but it is also associated with insufficient energy supply to the cows and some specific diseases, e.g., pasture bloat [32]. 

Welfare assessment of cows in rapidly expanding small holder dairy farms on a routine basis is important for providing feedback to the stakeholders for corrective interventions. Indicator-based welfare assessment of the cows in small holder dairy farms has rarely been reported in South Asia, even though there are reports of assessment frameworks for captive wild animals in this region of the world [33,34]. A welfare assessment in Bangladesh [35] reported many indicators of poor welfare in dairy cows, including body condition, cleanliness, injuries, diarrhoea, respiratory distress, coughing, and nasal and ocular discharges. In order to fill this research gap, we undertook a study in a prominent milk-producing district of Bangladesh using selected parts of the Welfare Quality protocol for dairy cattle as the basic framework for assessment, modified as necessary because of the context of Asian small holder dairy farms. We hypothesized that welfare of cattle in the small holder farms was adequate and the farmers have adopted welfare science-based management methods, as we operated in a high milk producing area of the country. The objective of this investigation was to evaluate dairy cow welfare in smallholder farms in Bangladesh, most of which used zero-grazing feeding systems. Associations between welfare parameters were determined to aid in deciding which of the European Welfare Quality® protocol measures [6] were most suitable to assess welfare in such situations. 

## 2. Materials and Methods 

The study was conducted on seventy smallholder dairy farms on the banks of the Baral river in the Sirajgonj district of Bangladesh (24° 27′ N, 89° 43′ E), which is the major milk-producing district of the country. These farms mostly supply milk to the Bangladesh Milk Producers’ Cooperative Union Limited (BMPCUL), the largest and oldest dairy cooperative in Bangladesh. It was conducted from May to July 2016, when the mean maximum and minimum temperatures in this region were 34.6 °C and 25.2 °C, respectively, and mean annual rainfall was 1610 mm.

### 2.1. Data Collection and Processing

Four villages were selected for sampling because they had the biggest concentration of dairy farms in the region. Within villages, farms were selected at random and the farmers were informed of the visit by the veterinarian from BMPCUL. Each farm was visited for one day, starting just after morning milking. 

In small farms (defined as containing 7–10 cows) interviews (detailed below) were conducted with family members, usually husband and wife, and in larger farms, the permanently hired labourer was interviewed. The farmer/hired labourer (hereafter, ‘the farmer’) was rewarded for co-operating with free mineral mixture supplements, anthelmintic boluses, and management advice. The face-to-face interviews with the farmers used a questionnaire with multiple-choice and semi-closed questions to collect animal- and management-linked data related to welfare. Information about feeding (type and schedule), milk yield of the cows, frequency of milking, technique of milking (hand or machine), whether teats were washed with water before and after milking (yes, occasionally, or no), whether application of antiseptic on teats before and after milking was done (yes or no), frequency of removal of the faeces from the house, incidence of mastitis and dystocia, and the vaccination and deworming schedule was collected in the farmers’ questionnaire (Table 1). Milk yield data was collected from the farmers and cross-checked with the data available in the BMPCUL records. The type of flooring and floor cleanliness was assessed by visual inspection. Assessment of management parameters from records and conducting the questionnaire-based farmer survey was done by only one observer. 

A total of 700 cows were examined at the 70 farms, of which 86% were lactating and 14% dry. The proportion of farms with 14–18 cows was 38%, 10–13 cows 47%, and 7–10 cows 15%. In farms with more than 15 cows, 12 cows were selected at random, in farms with 10–14 cows, 10 cows were selected, and in farms with 9 or fewer cows all were examined. The cow-based observations and clinical examinations (Table 2) were conducted on manually restrained cows in each farm by the second assessor, who was trained in the assessment protocol through initial pilot studies in two farms before the commencement of the actual study. The cows were identified by the farmer by name and subjected to inspection, which was from the side (left or right, randomly chosen) and from behind. 

The clinical examination of the cows was conducted as follows: 

(1) Cows were visually observed for general demeanor and recorded as alert, dull or depressed, modifying the four-point scale developed by Danscher et al., [36]. 

(2) Clinical examination of the ocular conjunctival mucous membrane was then performed and assessed as moist and pink, moist and pale pink, or dry and white. 

(3) Teats were physically examined and categorised as normal, deformed, cracked or dry. 

(4) Rumen condition was visually assessed and described as distended, hollow or normal. 

(5) Nasal, ocular, and vulvar discharges in the cows were visually inspected and recorded as absent (score 0) or present (score 1). 

(6) Cows were inspected for laboured/abnormal respiration, diarrhoea, and ectoparasitism and scored as absent (score 0) or present (score 1).

(7) Body Condition Score (BCS) was assessed on a four-point scale, modified from the three-point scale of the Welfare Quality protocol, and scored to whole units [6]. The cows were viewed from the side in the tail head and loin areas and back and classified as: 0–very thin; 1–thin; 2–fat; 3–very fat/ obese (details are provided in Table 1). 

(8) Cleanliness of the cows was assessed by the visual inspection of the hindquarters, hind legs below the hock joints, flanks, udder, and teats. These parts were classified as dirty if an area of >15 cm^2^ was found contaminated with soil or manure [25]. Lameness was assessed by walking the cows on their usual walking surface and visually inspecting their gait from behind and the sides; it was then scored on a three-point scoring system used by Breuer et al., [37].

(9) Lesions on the skin of the cows were assessed by visually inspecting six regions of the body (neck, brisket, carpal joint, tarsal joint, flank, and tuber coxae) from a randomly chosen side, thus modifying the method used by Kielland et al., [38], who assessed both sides. The total number of cows with hairless patches and lesions of >15 cm^2^, estimated visually, was recorded. 

### 2.2. Farmers’ and Stock People’s Attitudes on Animal Welfare

The farmers’ understanding of animal welfare, as defined by the five freedoms of animal welfare [5] and their assessment indicators, was evaluated by interviewing them. The following closed questions were asked (yes or no): whether the farmer had heard of animal welfare (translated into the native language), and whether they thought the following were important for animal welfare: access to feed and water at all times; minimisation of pain, distress, and suffering; veterinary treatment; and comfortable housing.

## 3. Statistical Analysis

Analysis was conducted using the statistical package Minitab (Minitab^®^ version 17.1.0, Minitab Inc., State College, PA, USA). Milk yield was estimated by the farmer in L/d, and for BCS mean values for each farm were calculated. Basic descriptive statistics were used for calculation of the mean and the percentage of different variables (skin lesions, lameness, cleanliness). This data on lameness, skin lesions and cleanliness of different body regions were expressed at the farm level, as percentages of the assessed cows. A prevalence rate of diseases and/or clinical signs reported by the farmer was calculated based on questionnaire results. Spearman rank correlations were calculated between milk yield, BCS, and dirtiness evaluations (teat, udder, flank, hind limb, and hindquarter), as the variables were not distributed normally. The number of cows assessed as dirty or with lesions in the different body parts was assessed by a Chi-square goodness of fit test. Ordinal logistic regression was used to relate floor cleanliness (1—clean to 4—very dirty), faeces removal (1 or 2 times per day or once every 2 days), and type of floor (soil, brick, or mixed soil and brick) to health-related parameters scored on an ordinal scale with a logit link function.

A stepwise linear regression model (forward/backward) was created using variables potentially associated with milk yield: lameness, cleanliness of floor, type of floor, lesions on the skin over hind limbs, udder, flank, hock and knee joints, ocular discharge, nasal discharge, respiratory problems, vaccination, deworming, mastitis, teat condition, ectoparasitic infestation, dull coat, cleanliness of body regions (flank, udder, teats, hind quarter, and hind limbs) as factors, and BCS as a covariate. Milk yield data was transformed to log_10_ to ensure that residuals were normally distributed (*p* = 0.54) by the Anderson Darling test. An analysis of variance (ANOVA) related milk yield to the number of cows with mastitis per farm. A stepwise linear regression mixed model was made of the association of these variables with BCS. Residuals were again normally distributed (*p* = 0.64) by the Anderson Darling test. Finally, a principal component analysis was undertaken of the measures specified above, using a correlation matrix, and presented as a loading plot.

## 4. Results

### 4.1. Systems of Dairy Farming Used 

All cows were of the Australian Holstein–Zebu cross genotype, this being most common in the area. All farms employed the ‘cut and carry’ system of feeding fresh grass, with some use of straw, hay and concentrate, mainly oilseed cakes. This was offered in tie stalls in the open air, without any building structure, usually adjacent to a loafing area. All herds were hand-milked twice a day in the cows’ stalls. Teats were sometimes cleaned before milking with water at body temperature, and occasional application of an antiseptic. No antiseptic treatment was used after milking. 

### 4.2. Milk Production and BCS

The mean milk yield, averaged across farms, was 10.3 L/d (SEM 0.259, range 6.3–14.2) and mean BCS was 1.96 (SEM 0.030, range 1.4–2.4). The distribution of mean adjusted milk yield/cow/d for all farms was normal. BCS, which necessarily had a discontinuous distribution, approximated a normal distribution (*p* = 0.03). 

### 4.3. Factors Affecting Milk Yield

The stepwise regression of milk yield against health, building and BCS parameters yielded significant negative associations with proportion of cows with hind limb lesions (*p* < 0.001), and mastitis (*p* = 0.003), and a moderate r^2^. Farms with cows with a high milk yield had fewer limb lesions and less mastitis (equation 1). Cows with mastitis had a reduced milk yield by on average 1.06 L/d (no mastitis 10.8, 10% of cows with mastitis 9.7, 20% of cows with mastitis 8.7 L/d, SED 0.371; *p* = 0.02).

Milk yield = 1.10 − 0.00186 (± 0.000529) hind limb lesions − 0.00448 (± 0.00141) mastitis; r^2^ adj. 23.2% (1)

where milk yield is log_10_ yield in L/d, the prevalence of hind limb lesions, and the incidence of mastitis were measured in % of cows in each farm.

**Figure 1:**
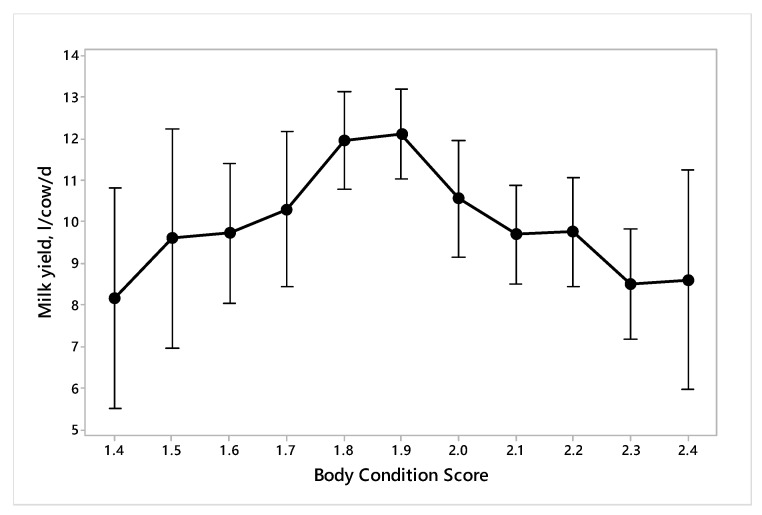
Change in milk yield with body condition score in dairy cows (n = 700) in 70 dairy farms assessed for the welfare of Australian–Zebu cross-bred dairy cattle in different areas of Sirajgonj district, Bangladesh.

The relation between BCS and milk yield was curvilinear (Figure 1). As mean body condition score increased up to 1.8–1.9, milk yield increased from approximately 8 to 12 L/d, an increase of approximately 8 L/unit BCS, then returned to 8 L/d as BCS increased further up to 2.4. 

### 4.4. Relationship between BCS and Health and Building Parameters

A stepwise regression of BCS against health parameters yielded significant positive associations with the proportion of cows with clean hind limbs (*p* = 0.006), clean flanks (*p* = 0.02), mastitis prevalence (*p* = 0.01), vaccinated (*p* = 0.002), a negative association with pale or white mucous membranes (*p* = 0.002) (equation 2), and a moderate r^2^.

BCS = c + 0.00398 (± 0.00141) hind limb cleanliness + 0.0470 (± 0.0203) flank cleanliness +0.00960 (± 0.00377) mastitis prevalence−0.00754 (±0.00234) pale/white mucous membrane. r^2^ adj = 28.6% (2)

where c = constant, which for vaccination levels 1 (never), 2 (occasional use) and 3 (routine) was 1.21, 1.46 and 1.55, respectively. Hind limb cleanliness, mastitis, mucous membrane colour, and flank cleanliness were measured in % of cows in each farm. 

### 4.5. Housing Facilities 

All farms had a shelter for their cows with a corrugated iron roof and stalls but no bedding, and a small loafing area. A total of 24.3% (n = 17) of farms had earthen floors in alleys between rows of stalls, 70.0% (n = 49) had brick floors and 5.7% (n = 4) had a combined soil and sand area and a brick area for loafing. Only 8.6% (n = 6) of the farms had maternity stalls into which pregnant cows were transferred in the last few days prior to parturition. In the remaining 91.4% (n = 64) of the farms, cows calved in their loafing area and in the alleys.

### 4.6. Cleanliness of Animals and House

Removal of faeces and cleaning of the stall floors on most of the farms was done once per day (61.4%, n = 43); 31.4% (n = 22) did this twice/day and for 7.1% (n = 5) it was done either occasionally or once every two days. The percentage of cows classified as dirty was higher in the regions of their hind quarter, lower hind limbs, flanks and udder (71.9% (n = 503), 67.6% (n = 473), 76.4 % (n = 535), 63.3% (n = 443), respectively) than for their teats 32.3 % (n = 226) [_ᵡ_^2^ = 137.2, *p* < 0.001]. Floor cleanliness showed a significant (*p* < 0.05) or close to significant (*p* < 0.10) relationship with lameness, hind limb cleanliness, udder cleanliness, body hair loss, respiratory problems, and mastitis (Table 3). In addition, the frequency of faeces removal demonstrated significant (*p* < 0.05) or close to significant (*p* < 0.10), negative relationship with hind limb cleanliness, neck lesions and deworming, and positive relationship with hair loss and mastitis. Furthermore, the type of flooring had significant (*p* < 0.05) or close to significant (*p* < 0.10) positive relationships with diarrhoea and mastitis, and negative relationships with flank cleanliness, hock lesions, ocular discharge, and deworming (Table 3).

### 4.7. Clinical Examination

The majority of the cows (98.0%, n = 686) were alert during observation and responded to external stimuli. The teats were normal in shape and healthy in 94.1% of cows (n = 659); however, in the remaining 41 cows (5.9%), at least one teat was cracked and deformed, generally due to a history of severe mastitis. The mucous membrane of the eyes was normal in 86.1% of cows (n = 603), pale pink in 13.7% (n = 96) and white in one cow. A total of 92.0% of cows (644) had a normal shape in the region of the rumen; 8.0% (n = 56) had a hollow rumen. Distended rumens were not observed. Regarding lameness prevalence, on 40 farms all cows scored 0, i.e. there was no evidence of lameness, 21 farms had one lame cow and 9 farms had two, with a prevalence of 5.6% lame of the total 700 cows. Of the lame cows, 27 were scored 1—lame, and 12 were scored 2—severely lame. Regarding skin lesions, more injuries to the carpal and tarsal regions were observed than to the neck, brisket, flank and tuber coxae, at farm level (farms: _ᵡ_^2^ = 12.6, *p* = 0.03; cows: _ᵡ_^2^ = 59.0, *p* < 0.0001) (Table 4).

### 4.8. Health Management and Status

Most farmers did not routinely vaccinate their stock, which is potentially necessary to control against FMD, black quarter, and anthrax in this region (regular vaccination 8.6%, n = 6; occasional vaccination 28.6%, n = 20; no vaccination 62.9%, n = 54). More farmers used anthelmintics, but only 24.3% (n = 17) used them regularly, 44.3% (n = 31) occasionally and 31.4% (n = 22) never used them. The most common diseases recorded were mastitis, ectoparasitism, and diarrhoea. Nasal discharge was the most common of all the discharge sites examined. 

### 4.9. Principal Components in the Welfare Parameters

**Figure 2:**
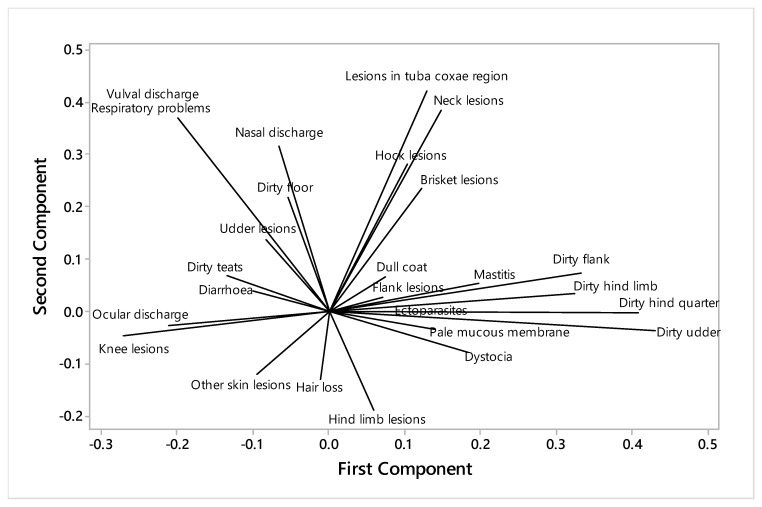
Principal component analysis of health and cleanliness factors of 700 cows in 70 herds in Sirajgonj district, Bangladesh.

Two main factors emerged from the PCA of welfare parameters measured on the farms, with Eigenvalues of 3.00 (component 1) and 2.58 (component 2) (Figure 2). The first factor reflected mainly differences in the cleanliness measurements, in which teat cleanliness was antagonistic to other cleanliness measures, confirmed by negative correlations with that for the udder (Correlation Coefficient (CC) −0.29, *p* = 0.01) and a tendency for the flank (CC −0.20, *p* = 0.09). The second factor appeared to relate to lesion sites, with hair loss, other skin lesions, and hind limb lesions being antagonistic to the neck and tuba coxae lesions.

### 4.10. Farmers’ Perspectives on Animal Welfare 

None of the farmers had heard of the term ‘animal welfare’. However, farmers in 94.3% (n = 66) and 72.9% (n = 51) of the dairy farms, respectively, agreed that cows should have ready access to feed and water. In 91.4% (n = 64) of the farms observed, farmers supported the requirement for alleviating unnecessary pain and suffering of the cattle, as well 87.6% (n = 27) of the farmers supported the provision of immediate veterinary assistance when required. However, in only 68.5% (n = 48) of the farms, farmers agreed that sufficient housing space with adequate facilities should be provided to allow the expression of normal behaviour patterns in the cows. In addition to the information on the daily milk yield of the cows, the farmers reported that the prevalences of mastitis and dystocia were 3.7% and 1.1%, respectively.

## 5. Discussion

This study is the first attempt to survey some resource and cow-based welfare parameters in dairy cows in smallholder farms in Bangladesh. Basic smallholder dairy farm levels of health, production, management, and welfare indicators were studied. The results potentially provide a tool not only for assessment, but also for consultancy and decision making to improve dairy cow welfare in different management systems of small holder dairy systems in developing countries. The Welfare Quality protocol [6] was the basic framework utilised in this study, and, with some modifications in the scoring of parameters, it was suited to local conditions and animals. 

### 5.1. Milk Yield and its Affecting Factors

The mean daily milk yield per lactating cow on farms in this study was low at just 10.3 L/d. Although there is an acknowledged influence of lactation stage and parity in daily milk [39], these factors were not included in our data set because farmers did not have this information. This yield is similar to previous reports from Bangladesh of 10.4 L/d/cow in high yielding cows [40], but higher than the reported average milk yield of 6.02 L/d/cow for cross-bred cows [41]. 

### 5.2. BCS and its Relationship with Health and Building Factors

We modified the three-point body condition scoring system of the Welfare Quality^®^ protocol to a four-point one, as our pilot studies revealed both very thin and thin cows and also fat and very fat/obese cows. The average milk yield had a curvilinear relationship with BCS. Milk yield of cows is also dependent on a combination of factors, including stage of lactation, breed, parity, and mastitis status [42]. At any one point in time, BCS and milk production curves are mirror images, with cows producing the most milk experiencing the lowest BCS, in early lactation. Furthermore, the effect of BCS on milk production is not a linear relationship [43], but a combination of genotypic and phenotypic effects. Previous studies have shown that cows in poor condition have reduced milk yield, in a linear relationship [12,44,45], and also poor reproductive performance [43]. In our study, the relationship was not linear and the initial increase and then decrease may be presumed to derive from benefits to milk production from increasing body reserves above a certain level, but also the fact that late lactation cows tend to have low milk yields and high BCS. The latter indicates that a high BCS may be beneficial, even though associated with low milk yield, as it provides reserves for subsequent milk production. BCS generally decreases in early lactation when nutrient intake does not meet the requirements for milk production, and then is restored in the later part of lactation, when milk yields have declined and feed intake increased. BCS also improves faster in primiparous cows than multiparous cows and the ones that are of lower genetic potential [46]. Gergovska et al., [47] indicated that on a lactation basis Friesian and Brown Swiss cows that lost more body condition had the highest milk yields and an optimal lactation duration.

The increase in cow cleanliness with BCS may derive from a general management factor: farmers feeding their cows well may also provide better facilities that keep the cows cleaner. It is also possible that within a farm, the cows with higher BCS are more dominant and able to gain access to the cleaner lying areas. The increase in mastitis with BCS may be because cows with excessively high BCS are prone to metabolic disorders and reduced milk yield [48,49]. Valde et al., [50] found that cows with a higher mean BCS around calving had increased rates of udder infections at the herd level. The negative association between milk yield and mastitis incidence probably reflects the impact of the disease on yield, although there could be a general management component: farmers achieving high milk yields are more likely to detect and treat mastitis early and provide cleaner conditions for their cows. An opposing effect may be that high milk yielding cows often have a significant energy deficit, which makes them prone to metabolic and reproductive problems [51].

### 5.3. Housing Facilities

Very few farms had a separate room for calving, even though parturition in alleys or loafing areas increases the risk of reproductive tract infections and calf disease, in particular, navel-ill [52]. Cows lay on floors with no bedding, on wet or moist brick floors with an uneven and abrasive surface and would therefore be expected to have an increased risk of lameness and limb injuries [53,54]. Furthermore, an earthen floor with pooled urine will lead to cows having dirty coats. Cows walking through excreta also have to alter their gait while walking [20]. 

### 5.4. Cleanliness of House and Animals

The cleanliness of cows and their housing is dependent on many factors, including stocking density, floor type, bedding materials, type of shelter, temperature, and humidity levels [55]. Frequency of cleaning the floor has a direct relationship with cleanliness [56], and in our study removal of faeces and cleaning of the stall floors were done only once or twice per day and on some farms less than once per day. The hindquarter, flank, tail, and udder are most likely to become dirty from manure and loose/diarrhoeic faeces. Similar to other studies, we also found some associations between dirty body regions and other housing (flooring) and management factors (cleanliness) [57,58,59]. However, several of the relationships listed in Table 3 have probabilities between 0.05 and 0.1 so caution is required in interpreting these. The udder and the teats are affected by the cleanliness and dryness of the stall surface, but teat cleanliness is also affected by the milking procedure. To ensure hygienic milk withdrawal, teats are usually cleaned before every milking [60]; thus, in our study, teats were cleaner than other body parts. 

### 5.5. Clinical Examinations

The majority of the cows in our study were alert with normal rumen shape and mucous membrane of eyes, and relatively few cows showing mild to moderate lameness during the examination. Hristov et al., [61] suggested that lameness is the major welfare problem for the dairy cow. Lameness has not only a major impact on welfare, it is also associated with poor performance and production [62,63]. The prevalence of lameness in our study was less than reported for loose-housed cows in more intensive dairy systems, where for example it has been reported that half the cows go lame in one year and 20% are lame at any one time [5]. In another study, the prevalence of herd lameness was estimated at 22% [64]. Estimates of the incidence of lameness in dairy cows vary considerably, ranging from 5.5 to 65% [65,66]. However, the prevalence of lameness in our study was similar to that previously reported for small farms in Tunisia [67]. The reason for the low prevalence of lameness could be the smaller concentrate ration available to the cows, in comparison to the western countries. This might reduce the occurrence of metabolic acidosis causing laminitis. Moreover, the scale of milk production is quite low in smallholder dairy units in comparison to the large-scale intensive dairy units in the West that have the highest incidence of lameness [68]. If the recent trend towards higher milk yields per cow continues lameness prevalence might increase in smallholder units, even if they use cross-bred cattle. Adverse effects of lameness on the productivity and fertility of cross-bred cows have been detected through changes in oestrus behaviour [69]. Milk production is often reduced in lame cows due to loss in body condition and also because of pain experienced during prolonged standing during the milking process [70].

### 5.6. Skin Lesions on Different Body Parts

The injuries to different parts of the body that were recorded in this study appeared linked to the different risk factors arising from the type of housing environment. The examined body protuberances were the parts on which maximum pressure is exerted when lying down. Injuries to the tarsal and carpal areas were common, probably caused by the rough and pot-holed brick floors. Similar injuries have been described in other studies [10,71]. These lesions cause pain [72] and demonstrate the close relationship between improperly designed or maintained animal facilities and the number of cows that are lame or injured [73] or have hock lesions [74,75]. Of all the health parameters measured in our study, it was the prevalence of limb lesions that related to milk output of the cows. The pain caused by the lesions during lying and standing may have prevented afflicted cows from resting normally. It is also possible that the relationship was not causal, and the farmers that did not provide comfortable facilities for their cows also did not manage them well to achieve high milk yields, in particular providing a nutritious diet. 

In addition to the floor type, another key predisposing factor to external body injuries is the restrictiveness of the housing structures, which may constrain the cows’ normal behaviour [37]. An improperly designed stall could make rising and lying behaviours difficult and result in more injuries to the hocks. Other lesions detected in the areas of the body subjected to pressure during feeding times, such as the brisket and neck, are in agreement with previous reports [37,76]. 

### 5.7. Health Management and Status

The most common diseases recorded were mastitis and ectoparasites, with diarrhoea also being common. Mastitis is one of the most important health problems in dairy cattle, with a major impact on welfare [77,78]. Ectoparasite infestation in cows, although being the second most common disease recorded, did not have a significant association with milk production, unlike mastitis. This might not encourage farmers to treat their cows with parasiticides. However, ectoparasitism decreases the BCS of the cows, and the latter in turn might affect the milk yield as an association between BCS and milk yield was found in the present study.

The principal component analysis demonstrated that teat cleanliness was antagonistic to other cleanliness measures, in particular, a negative correlation with udder cleanliness. This could be because dirty udders and flanks make the farmers clean the teats. The second factor seemed to relate to lesion sites, with hair loss, other skin lesions, and hind limb lesions being antagonistic to the neck and tuba coxae lesions.

### 5.8. Farmers’ Understanding of Animal Welfare

Overall, the farmers were not familiar with the term ‘animal welfare’, but they demonstrated that they believed that animal suffering and its alleviation have important relationships with animal comfort. Our findings are in accordance with a previously conducted similar study [79]. The few farmers who did not support the need for the alleviation of animal pain and suffering, as well as provision for animal comfort, were found to be better informed on factors that contribute to the improvement of production. Farmers therefore need training to understand basic concepts in animal welfare.

## 6. Conclusions

This study has indicated that some aspects of the Welfare Quality^®^ Assessment Protocol for cattle could be put in practice and implemented, with some modifications, for small scale production systems in Bangladesh. Welfare assessment using a full-scale version of the protocol should be explored in future studies. The study concluded that some housing and management factors were associated with BCS and milk yields in dairy cows. Milk production varied with BCS in a relationship that reflects the complexity of the lactation cycle in dairy cows. The study revealed that these dairy farming systems were associated with important animal welfare problems of injury and dirtiness in different parts of the body, which were related to reduced milk production. There was also evidence of inadequate use of vaccines and anthelmintics, which is likely to have significant impact on the cows’ welfare. The farmers in these smallholder units had little perception of animal welfare, and there was less recognition of the importance of good housing systems than other aspects of providing for the welfare of cows. There is a need to expose smallholder dairy farmers to training in good animal welfare practices, including appropriate housing designs, cleanliness of the house, and emphasizing the relationship between good animal welfare and productivity. Development of a countrywide framework for routine welfare assessment of small holder dairy farms could help in the identification and ameliorations of welfare problems.

## Figures and Tables

**Table 1:** Parameters measurement and methodology of data collection for the assessment of milk yield and health of the selected dairy cows based on management parameters used in the Welfare Quality^®^ assessment protocol (Welfare Quality^®^, 2009).

Parameters	Measurement Type	Description/Measures
Milk yield	Questionnaire and farm records	Average milk yield (l) / cow / day
House management	House-based measures; Questionnaire and direct observation	Flooring type (1: soil / 2: brick / 3: combined of soil and brick); frequency of faeces removal from the house (<1x/d, 1x/d or 2x/d) and floor cleanliness (1–clean, 2–mildly dirty, 3–moderately dirty, 4-very dirty)
Mastitis incidence	Questionnaire	Number of cows having suffered with an udder infection (clinical mastitis) during the last 12 months
Dystocia incidence	Questionnaire	Number of calvings where major assistance was required during the last 12 months.
Vaccination schedule	Questionnaire	Use of vaccines against important diseases (FMD, anthrax, black quarter); classified as never used, occasionally used, routinely used
Deworming schedule	Questionnaire	Use of anthelmintics; classified as 1, 2, or 3 times/year

**Table 2:** Parameters measurement and methodology of data collection for the assessment of health of the selected dairy cows based on animal parameters used in the Welfare Quality^®^ assessment protocol (Welfare Quality^®^, 2009).

Parameters	Description/Measures
Clinical examination	General appearance (alert/dull/depressed); mucous membrane of eye conjunctiva (moist and pink/moist and pale pink/dry and white); teat condition (normal/deformed/cracked/dry), rumen condition (distended, hollow or normal).
Body condition score	Visual appraisal by the assessor of individual BCS using a four-point scale, scored to whole units: 1–very thin (cavitation of tail head, depression at the tuber coxae region, transverse vertebral processes ends clearly visible, tail head, tuber coxae, spine and ribs visible) 2–thin (slight cavitation of tail head, flatness in the tuber coxae region, transverse vertebral processes ends clearly visible, spine and ribs visible but tail head not clearly visible); 3–fat (no cavitation around tail head but no folds of fatty tissue present, flatness in the tuber coxae region, ends of transverse vertebral processes slightly visible, ribs not visible); 4–very fat/ obese (no cavitation around tail head with presence of fatty tissue folds, convexity between the spine and tuber coxae, transverse vertebral processes not visible, extensive areas of fat under the skin).
Cleanliness	The hindquarter, lower hind leg (hock), flank, udder , and teats were inspected to assess cleanliness. Cows were classified as clean if there was no or only minor contamination (<15 cm^2^) with either soil or manure, otherwise they were classified as dirty.
Lameness	The cows were assessed from behind and from the side when walking on a surface on which they normally walked. A three-point scoring system was used (Breuer et al., 2000) 0 = not lame, timing of steps and weight-bearing equal on all 4 feet; 1 = lame, imperfect temporal rhythm in stride, creating a limp; and 2 = severely lame, strong reluctance to bear weight on one limb, or more than one limb affected.
Skin lesions	Six body regions of cows (neck, brisket, carpal and tarsal joint, flank and tuber coxae) were evaluated from one side (randomly chosen). In each region, the number of cows with hairless patches and lesions/swellings of >15 cm^2^ were recorded.
Nasal discharge	Scale: 0: Little or no evidence of discharge 1: Evidence of clearly visible flow/discharge from the nostrils; transparent to yellow/green and often of thick consistency
Ocular discharge	Scale: 0: Little or no evidence of discharge, or 1: Evidence of clearly visible flow/discharge (wet or dry) from the eye, at least 3 cm long
Vulval discharge	Scale: 0: Little or no evidence of discharge 1: Evidence of purulent effluent from the vulva, including on the underside of the tail
Laboured respiration	Scale: 0: No evidence of abnormal respiration 1: Evidence of deep and laboured respiration; expiration usually accompanied by pronounced sound
Diarrhoea	Scale: 0: Little or no evidence of abnormal consistency of faeces 1: Evidence of loose watery faeces around the tail
Ectoparasitic infestation	Close inspection, including with a hair comb to find any mites or ticks

**Table 3:** Significant (*p* < 0.05) or close to significant (*p* >0.05 < 0.10) health variables of cows related to floor cleanliness (1—clean to 4—very dirty), frequency of faeces removal and type of floor by ordinal logistic regression

Predictor	Coefficient	SE Coefficient	*p*-Value	Odds Ratio	95% CI
Floor cleanliness (1 clean to 4 very dirty)					
Lameness	−0.103	0.059	0.069	0.90	0.80–1.01
Hind limb cleanliness	0.041	0.021	0.053	1.04	1.0–1.09
Udder	0.064	0.027	0.022	1.07	1.01–1.13
Hair loss	−0.153	0.060	0.011	0.86	0.76–0.97
Respiratory problem	0.320	0.185	0.084	1.38	0.96–1.98
Mastitis	0.129	0.061	0.036	1.14	1.01–1.28
Frequency of faeces removal (1: <1x/d, 2: 1x/d, 3: 2x/d)					
Hind limb cleanliness	−0.048	0.026	0.066	0.95	0.91–1.00
Neck lesion	−0.258	0.095	0.007	0.77	0.64–0.93
Hair loss	0.106	0.062	0.080	1.11	0.98–1.26
Deworming	−1.244	0.687	0.070	0.29	0.07–1.11
Mastitis	0.157	0.065	0.016	1.17	1.03–1.33
Floor type (1: earth floor, 2: brick floor)					
Flank cleanliness	−0.053	0.031	0.084	0.95	0.89–1.01
Hock lesion	−0.126	0.052	0.016	0.88	0.79–0.98
Ocular discharge	−0.514	0.176	0.004	0.60	0.42–0.85
Diarrhoea	0.212	0.100	0.034	1.24	1.02–1.51
Deworming	−2.186	1.063	0.040	0.11	0.01–0.90
Vaccination	2.609	1.444	0.071	13.60	0.80–230.67
Mastitis	0.323	0.110	0.003	1.38	1.11–1.72

**Table 4:** Prevalence of lesions on various parts of the body of 700 cows examined in 70 dairy units evaluated for welfare of dairy cattle in the Sirajgonj district, Bangladesh

Body Regions with Injury	Farms		Cows	
	Number of Farms (n = 70)	Percentage (%)	Number of Cows (n = 700)	Percentage (%)
Carpal joint	39	55.71	64	9.14
Tarsal joint	36	51.43	79	11.29
Neck	31	44.29	39	5.57
Brisket	24	34.29	30	4.29
Flank	20	28.57	23	3.29
Tuber coxae	19	27.14	27	3.86
